# Supplementary material for: Association between oral microbiome and seven types of cancers in East Asian population: a two-sample Mendelian randomization analysis
Source: Front Mol Biosci. 2023 Nov 21;10:1327893. doi: 10.3389/fmolb.2023.1327893 (PMC10702768; doi:10.3389/fmolb.2023.1327893)
Supplement: Supplementary file 1 [file DataSheet1.ZIP › Supplementary Materials/MR plots of saliva/saliva═╝/Lung Cancer/pheno.2778_to_lung cancer_leave_one_out.pdf]

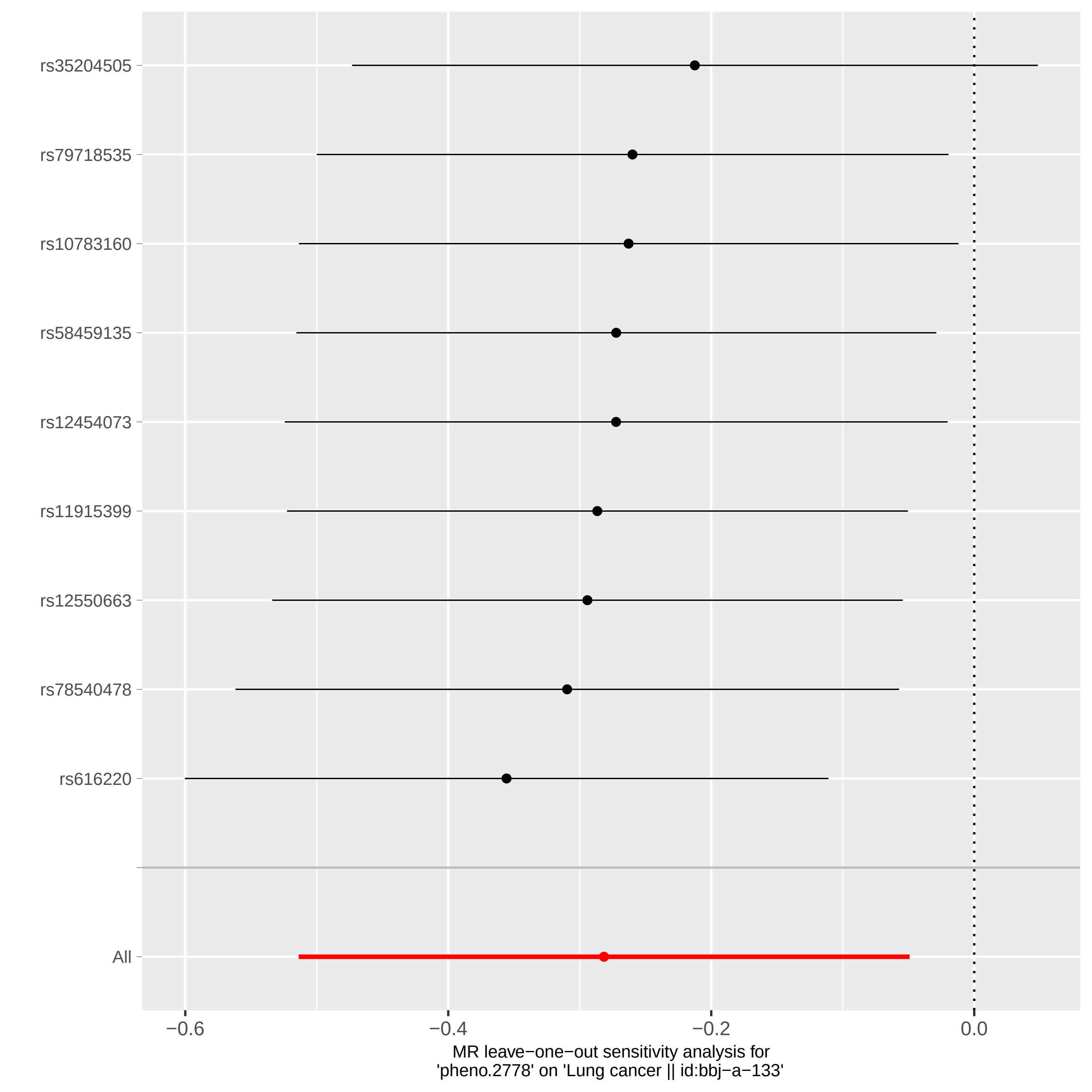

rs35204505
rs79718535
rs10783160
rs58459135
rs12454073
rs11915399
rs12550663
rs78540478
rs616220
All
−0.6
−0.4
−0.2
0.0
MR leave−one−out sensitivity analysis for
'pheno.2778' on 'Lung cancer || id:bbj−a−133'
